# Prognostic Significance of Cell-Free DNA Derived 5-Hydroxymethylcytosine Signatures in Newly Diagnosed Multiple Myeloma

**DOI:** 10.1200/PO-25-01121

**Published:** 2026-07-23

**Authors:** Zhou Zhang, Benjamin Derman, Bei Wang, Krissana Kowitwanich, John Cursio, Lu Gao, Daniel Appelbaum, Chuan He, Andrzej Jakubowiak, Wei Zhang, Brian C. Chiu

**Affiliations:** ^1^ Department of Preventive Medicine, Northwestern University Feinberg School of Medicine, Chicago, IL; ^2^ Section of Hematology/Oncology, Department of Medicine, The University of Chicago, Chicago, IL; ^3^ Department of Public Health Sciences, The University of Chicago, Chicago, IL; ^4^ Department of Chemistry, The University of Chicago, Chicago, IL; ^5^ Department of Radiology, The University of Chicago, Chicago, IL; ^6^ Department of Biochemistry and Molecular Biology, Institute for Biophysical Dynamics and Howard Hughes Medical Institute, The University of Chicago, Chicago, IL

## Abstract

**PURPOSE:**

While survival outcomes in multiple myeloma (MM) have improved with contemporary combination therapies, predicting disease trajectories for individual patients at diagnosis remains a significant challenge. We investigate the prognostic value of a noninvasive biomarker—cell-free DNA (cfDNA)‑derived 5-hydroxymethylcytosine (5hmC) signature—in newly diagnosed MM, aiming to improve risk stratification at diagnosis.

**MATERIALS AND METHODS:**

In this prospective cohort study, 321 patients with newly diagnosed MM were enrolled between 2010 and 2017, with follow-up through 2022. We profiled genome-wide 5hmC modifications (gene bodies) in cfDNA collected at diagnosis. We applied elastic net regularization to a Cox proportional hazards model to identify 5hmC signatures associated with overall survival (OS) and progression-free survival (PFS). A weighted prognostic score (wp-score), based on 18 key 5hmC-modified genes, was developed through machine learning and validated in the validation set, controlling for clinical prognostic factors.

**RESULTS:**

During a median follow-up of 70.5 months, 127 deaths occurred. The cfDNA 5hmC at diagnosis reflected the 5hmC in bone marrow‑derived tumor cells and differed across clinical subgroups. The wp-score showed strong prognostic ability for OS (hazard ratio [HR], 2.9 [95% CI, 1.7 to 4.9]; *P* < .0001) and PFS (HR, 1.8 [95% CI, 1.3 to 2.5] *P* = .00014) in the validation set, controlling for known prognostic factors, including stage, lactate dehydrogenase levels, and treatment type. The wp-score calculated at diagnosis remained associated with OS and PFS at 24-, 48-, and 72-month follow-up periods.

**CONCLUSION:**

This prospective study suggests that cfDNA-derived 5hmC signatures at diagnosis provide independent prognostic information for OS and PFS in MM. These findings support further investigation and independent validation of noninvasive epigenomic biomarkers within contemporary MM risk stratification frameworks.

## INTRODUCTION

Multiple myeloma (MM), the second most common hematologic malignancy,^1^ is a heterogeneous disease with marked variability in clinical course and survival.^2^ Despite advances in therapy, including multidrug combinations and autologous stem-cell transplant (ASCT),^3^ median survival remains at approximately 6 years.^4^ Predicting patient outcomes remains challenging. Risk stratification at diagnosis is essential, but current prognostic factors have important limitations and are not applicable to all patients.^5^ Laboratory measures, such as β_2_-microglobulin,^6^ free light chain ratio,^7^ and lactate dehydrogenase (LDH) level^8^ explain < 20% of clinical variability. High-risk cytogenetics^9^—including t(14;16), t(14;20), gain of 1q, and del(17p)—occur in only approximately 15%-30% of patients.^10^ Although gene expression profiling^11^ offers greater specificity,^12^ its routine clinical use is limited by cost, invasive bone marrow sampling, and challenges in isolating CD138+ tumor cells from genetically heterogeneous marrow samples.^13-15^

**CONTEXT**

**Key Objective**
To evaluate the prognostic value of cell-free DNA (cfDNA)-derived 5-hydroxymethylcytosine (5hmC) signatures in newly diagnosed multiple myeloma (MM) and to assess their ability to improve risk stratification at diagnosis.
**Knowledge Generated**
The cfDNA 5hmC signatures at diagnosis reflected those of bone marrow‑derived tumor DNA and differed across clinical subgroups. A weighted prognostic score based on 18 key 5hmC genes at the time of diagnosis was independently associated with both overall and progression-free survival and demonstrated consistent prognostic performance over multiple follow-up durations.
**Relevance**
The cfDNA-derived 5hmC signature represents a promising noninvasive prognostic biomarker in MM. Independent validation is warranted to define its clinical utility within contemporary risk stratification frameworks and evolving therapeutic paradigms.

These limitations underscore an urgent need for improving risk stratification at diagnosis. Aberrant epigenetic modifications play a critical role in the progression and aggressiveness of MM. Promoter hypermethylation of tumor suppressors—for example, *GPX3*, *RBP1, SPARC*, and *TGFBI*—has been associated with shorter overall survival (OS)^16^ and hypermethylation of *RASD1* correlates with resistance of MM to dexamethasone.^17^ However, most epigenetic studies overlooked 5-hydroxymethylcytosines (5hmC), a distinct epigenetic mark from the more common 5-methylcytosines (5mC), involved in gene activation and chromatin remodeling.^18,19^ The number of 5hmC-expressing cells is lower in plasma cell myeloma than in reactive plasma cell hyperplasia,^20^ and global 5hmC level is significantly reduced in MM cells, compared with normal plasma cells.^21,22^ In addition, specific hydroxymethylation has been associated with MM cell proliferation.^23^ A recent study showed that a lower global 5hmC level, but not 5mC, was associated with worse OS of MM.^22^ However, the clinical application of 5hmC in MM remains largely unknown.

The recent technical advances enable genome-wide 5hmC profiling in circulating cell-free DNA (cfDNA),^24-26^ a minimally invasive alternative to bone marrow biopsy. This liquid biopsy not only captures DNA from bone marrow‑derived plasma cells and extramedullary sites^27,28^ but also circumvents the pitfalls of heterogeneity in bone marrow sampling.^29^ In this prospective study, we used the 5hmC-Seal,^26^ a highly sensitive chemical labeling technology to profile genome-wide 5hmC in cfDNA from plasma samples of 321 newly diagnosed patients with MM. We hypothesize that 5hmC profiles in cfDNA at diagnosis correlate with key clinical prognostic features of MM and are predictive of survival outcomes. By integrating cfDNA-based 5hmC into current prognostic models, we aim to provide biologically informed risk stratification at diagnosis that could improve personalized management of patients with MM.

## MATERIALS AND METHODS

### Study Participants and Data Collection

The University of Chicago (UChicago) Multiple Myeloma Epidemiology Study (MMEP) prospectively enrolled adult patients aged 20 years and older with newly diagnosed MM at the UChicago Medical Center from 2010 to 2019. Informed consent was obtained from all participants. Blood samples and epidemiology questionnaire were collected at the time of enrollment. Comprehensive clinical, laboratory, and treatment data were extracted from electronic medical records. These data included details on treatment regimens, transplant status, and treatment outcomes (eg, response to treatment, progression, and relapse). Mortality data were ascertained using the National Death Index. The study was approved by the UChicago Institutional Review Board.

This report included 324 plasma samples collected between 2010 and 2017. We also obtained plasma-paired CD138+ tumor cells from bone marrow aspirates at diagnosis for 14 patients. After quality control,^25^ 5hmC data from 321 patients with MM were retained for analyses. Progressive disease was defined according to the International Myeloma Working Group (IMWG).^30^ Progression-free survival (PFS) was defined as the time from diagnosis to progression or death, and OS was defined as the time from diagnosis to death from any cause. Follow-up was through February 2022 (median: 70.48 months, range: 1.2 to 120.0 months).

### Sample Preparation, 5hmC-Seal Profiling, Sequencing, and Bioinformatics

Details for 5hmC-Seal library construction and data processing have been described previously.^24-26,31-37^ Briefly, the 5hmC-Seal data were processed using established pipelines for read trimming, alignment, and gene-level quantification, followed by normalization using DESeq2.^38^ Additional details and sequencing quality metrics are provided in Data Supplement Methods and Table S1.

### Correlation Between cfDNA and gDNA From CD138+ Plasma Cells

To assess the concordance between cfDNA and matched CD138+ plasma cell genomic DNA (gDNA), gene bodies were ranked by variability in 5hmC modification levels, and overlap was evaluated using permutation (10,000 iterations) with a hypergeometric test. Pearson correlation coefficients were calculated to quantify similarity of 5hmC patterns within and between individuals. Details are provided in Data Supplement Methods.

### Cellular Deconvolution

We estimated contributing cell sources in cfDNA using CIBERSORTx.^39^ A reference representing major immune cells (B cells, monocytes, NK cells, and CD4^+^ and CD8^+^ T cells) was derived from the study by Nakauchi et al.^40^ Estimated cell-type proportions were normalized to a sum of 100%. Differences between groups were assessed using Wilcoxon rank-sum test. Details are described in Data Supplement Methods.

### Association Between 5hmC in cfDNA and Clinical Prognostic Factors

The examined clinical factors included International Staging System (ISS) stage, serum LDH levels, estimated glomerular filtration rate (eGFR), serum free light chain ratios, frontline treatment regimens, and ASCT status. We used DESeq2 to identify 5hmC features associated with each clinical factor.^38^ For the ISS, stages II and III were combined into a single category (ie, higher stages), while stage 1 was the reference (ie, early-stage disease). The categorization of frontline treatments was based on the number of drugs used in combination (eg, doublet, triplet, quadruplet, or others). Owing to the limited number of patients receiving quadruplet therapy, these were grouped with the triplet treatments, with doublet therapy as the reference group. Continuous clinical variables such as LDH levels (normal [<240 U/L] vs. elevated [≥240 U/L]) and eGFR (≥60 mL/min *v* <60 mL/min) were categorized according to standard clinical thresholds.

### Development of a Weighted Prognostic Model for MM

Data Supplement, Figure S1, illustrates the overall study design. A total of 321 patients (alive, n = 194; death, n = 127) were randomly divided for training (n = 161; alive = 97, death = 64) and validation (n = 160; alive = 97, death = 63). Given the modest sample size, a 50:50 split was chosen to balance model training and independent validation, with no significant differences in age or sex between the sets.

To reduce dimensionality while retaining potential informative features, we applied a permissive screening step using Cox proportional hazards models adjusted for age and sex during training to identify candidates associated with survival (*P* < .05). Next, we applied elastic net regularization to multivariable Cox models using *glmnet*^41^ to identify the final panel for survival outcomes. Modeling was repeated across 100 resamplings, and features selected in ≥50% of iterations were retained to fit a final prognostic model. The weighted prognostic score (wp-score) was calculated as wp-score = $\frac{1}{1+e^{-\left(\beta_{0}+\sum \beta_{k}G_{k}\right)}}$, where $\beta_{0}$ represents the intercept, *β*_*k*_ the logistic regression coefficient, and *G*_*k*_ the normalized 5hmC level for feature *k*_*th*_. The wp-score was scaled from 0 to 1.

Survival differences were assessed using Kaplan-Meier (KM) survival curves with median wp-score stratification and log-rank tests. Prognostic accuracy of wp-score was evaluated using time-dependent receiver-operating characteristic (ROC) analysis, with AUC calculated at predefined time points for OS and PFS.

### Evaluation of Wp-Scores in Relation to Clinical Prognostic Indices and Estimated Cellular Contributions

The examined clinical prognostic factors included ISS, LDH, eGFR, treatment types, and transplantation status, in addition to the estimated cellular sources (eg, cell types >50% contribution or *P*-value <.05 between alive and deceased patients). To assess its independent prognostic significance, we constructed a multivariable Cox model (eg, a full model) incorporating the wp-score, established prognostic factors, and cellular sources in the pooled cohort. Stepwise regression based on the Akaike information criterion (AIC) was applied to derive an integrated predictive model for survival outcomes. LASSO was also used to evaluate the wp-score as an independent predictor. Model performance was compared with the wp-score within the same cohort. The proportional hazards assumption was evaluated using Schoenfeld residuals, with no evidence of violation.

### Pathway Analysis

To provide biological insights, we performed Kyoto Encyclopedia of Genes and Genomes (KEGG)^42^ pathway enrichment analysis using *clusterProfiler*.^43^ We considered pathways with at least 15 genes and a *P* value <.05 as statistically significant.^44^

## RESULTS

### Patient Characteristics

Among the 321 patients (Table 1), 127 deaths were recorded over a median follow-up period of 70.5 months. Most of the patients were European Americans (65.7%) with stage 1 disease, a normal LDH level, and eGFR ≥ 60 and were treated with triplet or quadruplet regimens (61.9%) and ASCT (81.6%). Compared with at-risk patients, deceased patients were more likely to be 60 years or older or African Americans with advanced disease (higher stage) and elevated LDH at diagnosis. They were also less likely to have received triplet or quadruplet therapies as part of their frontline treatment.

**TABLE 1.** Characteristics of the Study Participants, UChicago MMEP Study

Characteristic	Total (No. = 321, %)	At Risk (No. = 194, %)	Death (No. = 127, %)	*P*
Age				
<60	133 (41.4)^a^	91 (46.9)	42 (33.3)	.06
60-69	129 (40.2)	71 (36.6)	58 (46.0)	
≥70	58 (18.1)	32 (16.5)	26 (20.6)	
Race/ethnicity				
European American	211 (65.7)	134 (69.1)	77 (61.1)	.06
African American	92 (28.7)	47 (24.2)	45 (35.7)	
Others	13 (4.0)	10 (5.2)	3 (2.4)	
Sex				
Female	161 (50.2)	99 (51.0)	62 (49.2)	.84
Male	159 (49.8)	95 (49.0)	64 (50.8)	
Education				
Below college	68 (21.2)	39 (20.1)	29 (23.0)	.35
Some college or completed college	61 (19.0)	33 (17.0)	28 (22.2)	
Graduate or professional degree	113 (35.2)	73 (37.6)	40 (31.7)	
ISS				
1	147 (45.8)	102 (52.6)	45 (35.7)	.01
2	59 (18.4)	34 (17.5)	25 (19.8)	
3	18 (5.6)	6 (3.1)	12 (9.5)	
LDH levels (U/L)				
Normal (LDH <240)	187 (58.3)	127 (65.5)	60 (47.6)	.01
Elevated (LDH ≥240)	65 (20.2)	31 (16.0)	34 (27.0)	
Estimated GFR (mL/min/1.73 m^2^), median				
≥60	228 (71.0)	143 (73.7)	85 (67.5)	.21
<60	85 (26.5)	46 (23.7)	39 (31.0)	
Serum free light chain (kappa/lambda) ratio				
Normal (0.26-1.65)	98 (30.5)	62 (32.0)	36 (28.6)	.73
High (>1.65)	151 (47.0)	88 (45.4)	63 (50.0)	
Low (<0.26)	59 (18.4)	35 (18.0)	24 (19.0)	
Treatment type				.01
Doublet	71 (22.2)	34 (17.5)	37 (29.4)	
Triplet/quadruplet	198 (61.9)	133 (68.6)	65 (51.6)	
Others + NA	51 (15.9)	27 (13.9)	24 (19.0)	
ASCT				.42
No	51 (15.9)	28 (14.4)	23 (18.3)	
Yes	261 (81.6)	162 (83.5)	99 (78.6)	
High-risk cytogenetics				.89
No	133 (41.6)	89 (45.9)	44 (34.9)	
Yes	24 (7.5)	17 (8.8)	7 (5.6)	

Abbreviations: ASCT, autologous stem-cell transplantation; GFR, glomerular filtration rate; ISS, International Staging System; LDH, lactate dehydrogenase; NA, not available; MMEP, The Multiple Myeloma Epidemiology Study; UChicago, The University of Chicago.

^a^ Number may not sum to 100 because of missing value. *P* value was computed using Chi-square test for comparisons of categorical variables.

### 5hmC-Seal Data and Clinical Characteristics

Data Supplement (Figure S2A-C) shows the top 25 genes ranked by *P* value for three major clinical prognostic factors. Overall, we observed differential 5hmC (*P* < .01) between clinical subgroups across all factors evaluated. For example, 114 gene bodies showed differential 5hmC between patients with early-stage (ie, ISS stage I) and higher stage (stages II/III) disease. Additionally, 1,291 and 54 gene bodies showed differential 5hmC between patients with elevated versus normal LDH levels and eGFR < 60 versus ≥60, respectively.

### Cellular Deconvolution of cfDNA

**FIG 1.**
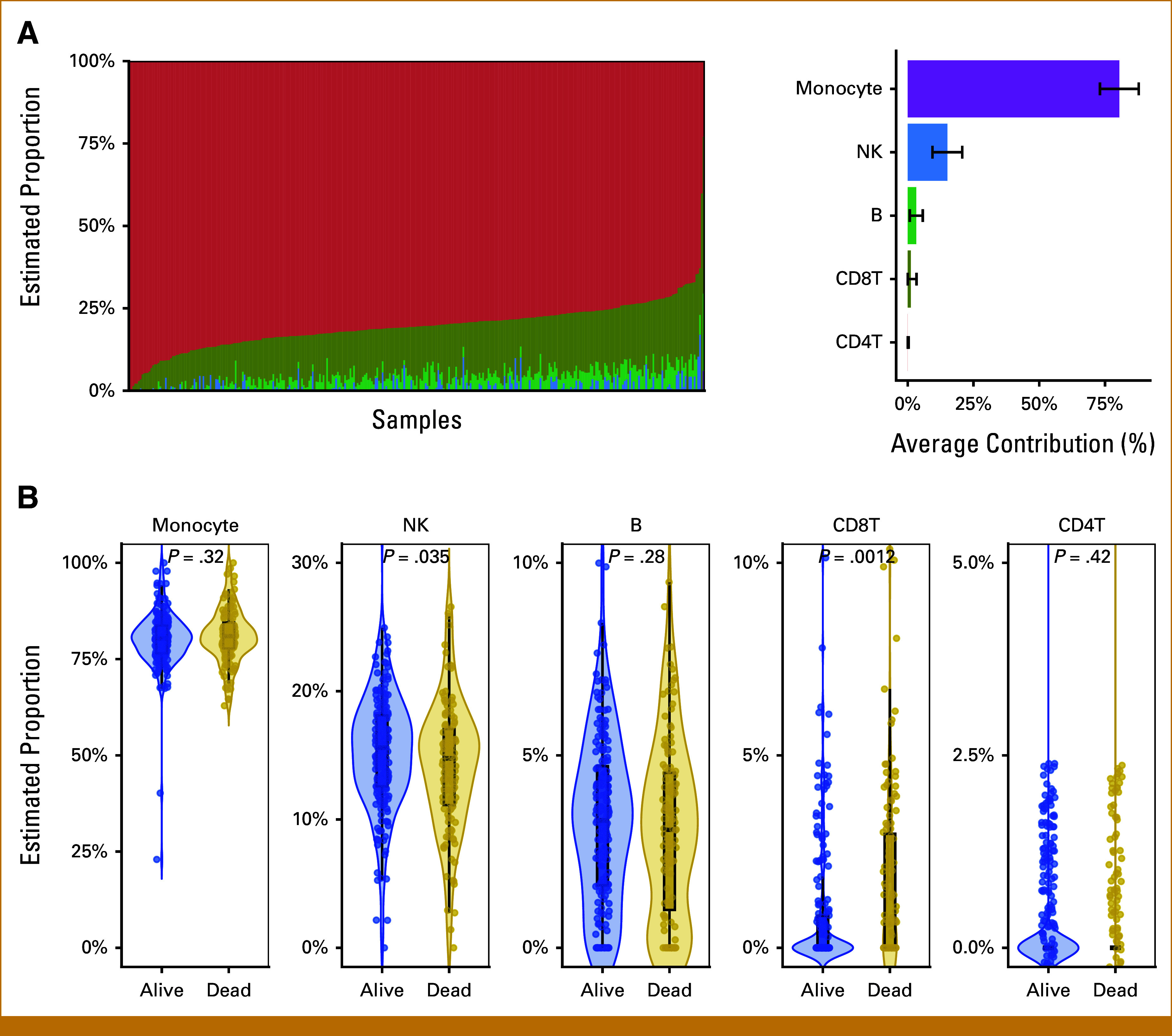
Cellular deconvolution of 5hmC in cfDNA. (A) Estimated cellular contributions from monocytes, NK T cells, B-lymphocytes (B cells), CD8T, and CD4T; (B) comparison of the contributions of each of the five immune cell types between alive and deceased patients at the time of diagnosis. *P* values were obtained from Wilcoxon rank-sum test. cfDNA, cell-free DNA; CD4T, CD4^+^ T-lymphocytes; CD8T, CD8^+^ T-lymphocytes; 5hmC, 5-hydroxymethylcytosine; NK, natural killer.

We used a cell-type‑specific 5hmC reference^40^ to perform cellular deconvolution and evaluate potential origins of cfDNA (Fig 1A). Of note, monocytes were the major source in cfDNA, accounting for an average contribution of 80.4%, followed by NK cells. The Wilcoxon rank-sum test showed that contributions of NK and CD8^+^ T cells at the time of diagnosis differed between at-risk patients and those later deceased. By contrast, the contributions from B cells, monocytes, and CD4^+^ T cells did not differ significantly between these two groups (Fig 1B).

### 5hmC in cfDNA Reflects Plasma Cell‑Derived gDNA Profiles

**FIG 2.**
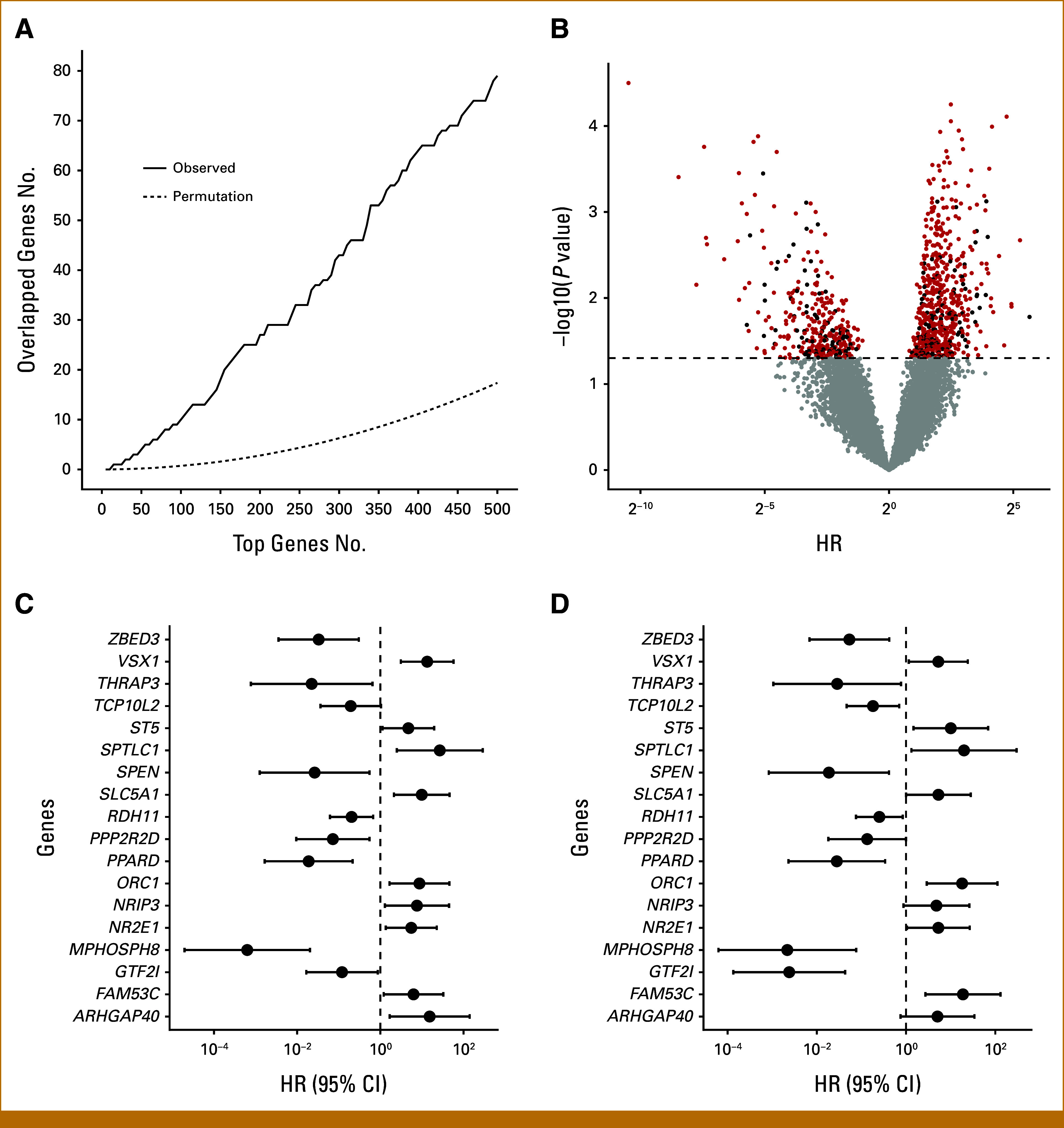
5hmC in cfDNA reflects the 5hmC in gDNA and demonstrates prognostic value. (A) The number of overlapping top variable genes was significantly higher than expected from a null distribution generated by permutation; (B) a total of 992 genes associated with OS in the training set (*P* < .05). Red dots indicate genes with associations in the same direction in the validation set; (C, D) forest plots illustrating the individual performance of the 18-gene markers in the training (C) and validation (D) sets. 5hmC, 5-hydroxymethylcytosine; cfDNA, cell-free DNA; HR, hazard ratio; OS, overall survival.

We found a significant overlap in gene bodies with high variability between cfDNA- and gDNA-derived 5hmC profiles (Fig 2A), based on a null distribution from permutation. Of the top 200 most variable features, 27 were shared in both cfDNA and gDNA. The within-patient correlation for these 27 gene bodies was stronger than between-patient comparisons, with mean Pearson r values of 0.865 and 0.826, respectively (t-test *P* value = .04), indicating that the cfDNA-derived 5hmC profiles reflected gDNA from CD138+ tumor cells.

### Prognostic Value of 5hmC-Based Models in cfDNA for MM

**FIG 3.**
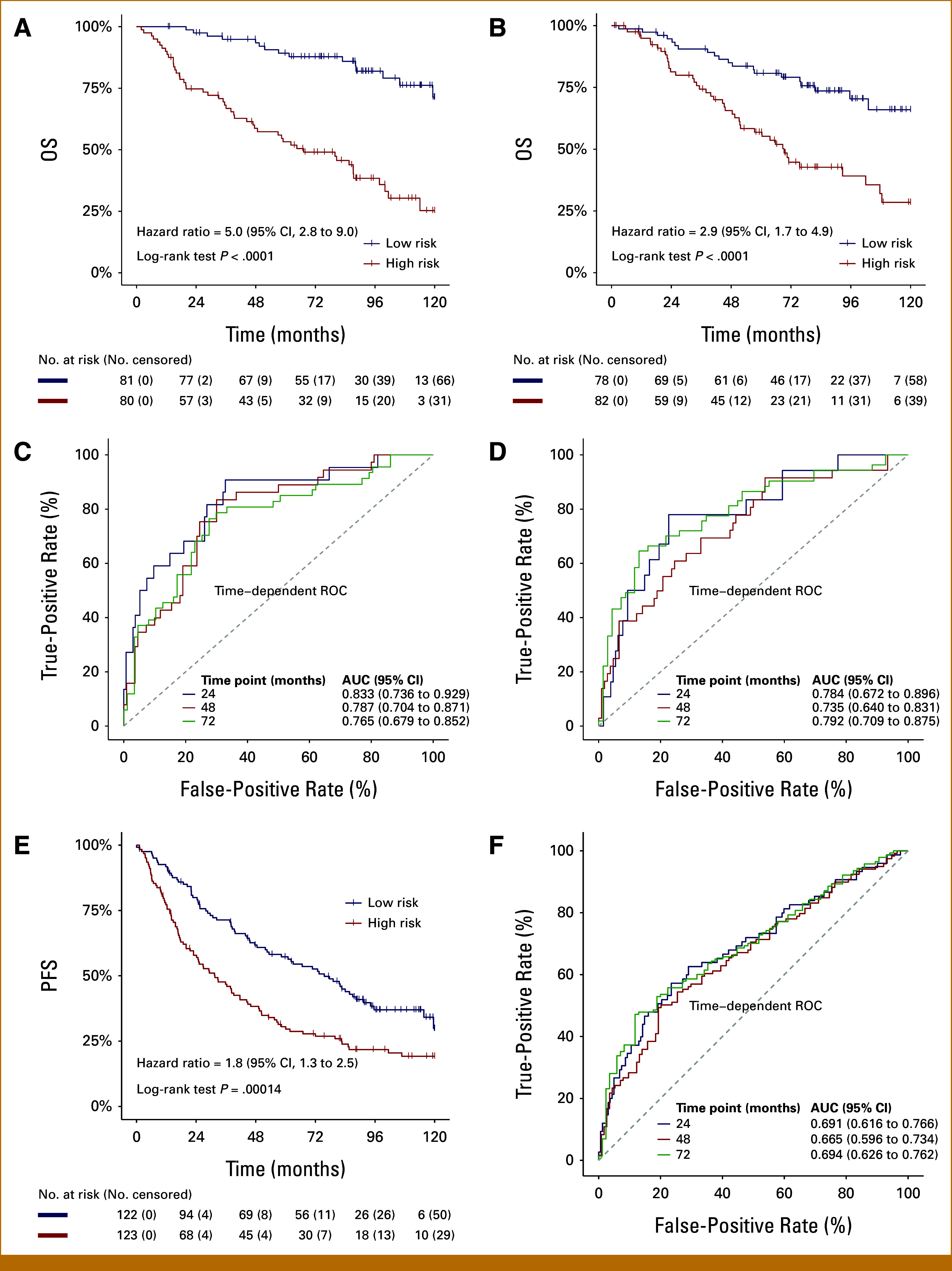
Prognostic value of 5hmC-based models in cfDNA for MM. (A,B) Performance of the 5hmC-based wp-score for OS in the training (A) and validation (B) sets; (C,D) time-dependent ROC analyses demonstrating robust prognostic performance of the wp-score for OS in the training (C) and validation (D) sets; (E) performance of the wp-score for PFS in the validation set; (F) time-dependent ROC analyses demonstrating prognostic performance of the wp-score for PFS in the validation set. 5hmC, 5-hydroxymethylcytosine; cfDNA, cell-free DNA; HR, hazard ratio; MM, multiple myeloma; OS, overall survival; PFS, progression-free survival; ROC, receiver-operating characteristic; wp-score, weighted prognostic score.

We identified 992 gene bodies showing a trend of association with OS in training (*P* < .05; ie, candidates for further analysis), of which 780 showed consistent directions in validation (Fig 2B). Feature selection yielded an 18-feature panel used to construct the wp-score (Figs 2C and 2D; Data Supplement, Tables S2 and S3). In the training set (deaths = 64), higher wp-scores were associated with worse OS (hazard ratio [HR], 5.0 [95% CI, 2.8 to 9.0]; Fig 3A). A similar trend was observed in the validation set (deaths = 63; HR, 2.9 [95% CI, 1.7 to 4.9]; Fig 3B). The prognostic value of the wp-score was consistent across different subgroups, for example, by race, sex, age group, and frontline therapy types (Data Supplement, Fig S3). Enrichment analysis of the 992 candidates showed involvement in key biological pathways (Data Supplement, Fig S4), including calcium signaling, neuroactive ligand-receptor interaction, and retrograde endocannabinoid signaling.

Time-dependent ROC analyses demonstrated consistent predictive performance of the wp-score, with AUCs of 0.833, 0.787, and 0.765 at 24, 48, and 72 months, respectively, in the training set, and 0.784, 0.735, and 0.792, respectively, in the validation set (Figs 3C and 3D).

Next, we assessed to what extent the wp-score developed for predicting OS had prognostic value for PFS. We found that higher wp-scores were associated with poorer PFS in the validation set (HR, 1.8 [95% CI, 1.3 to 2.5]; Fig 3E), with corresponding time-dependent AUCs of 0.691, 0.665, and 0.694 at 24, 48, and 72 months, respectively (Fig 3F).

### Independent Prognostic Significance of Wp-Scores

**FIG 4.**
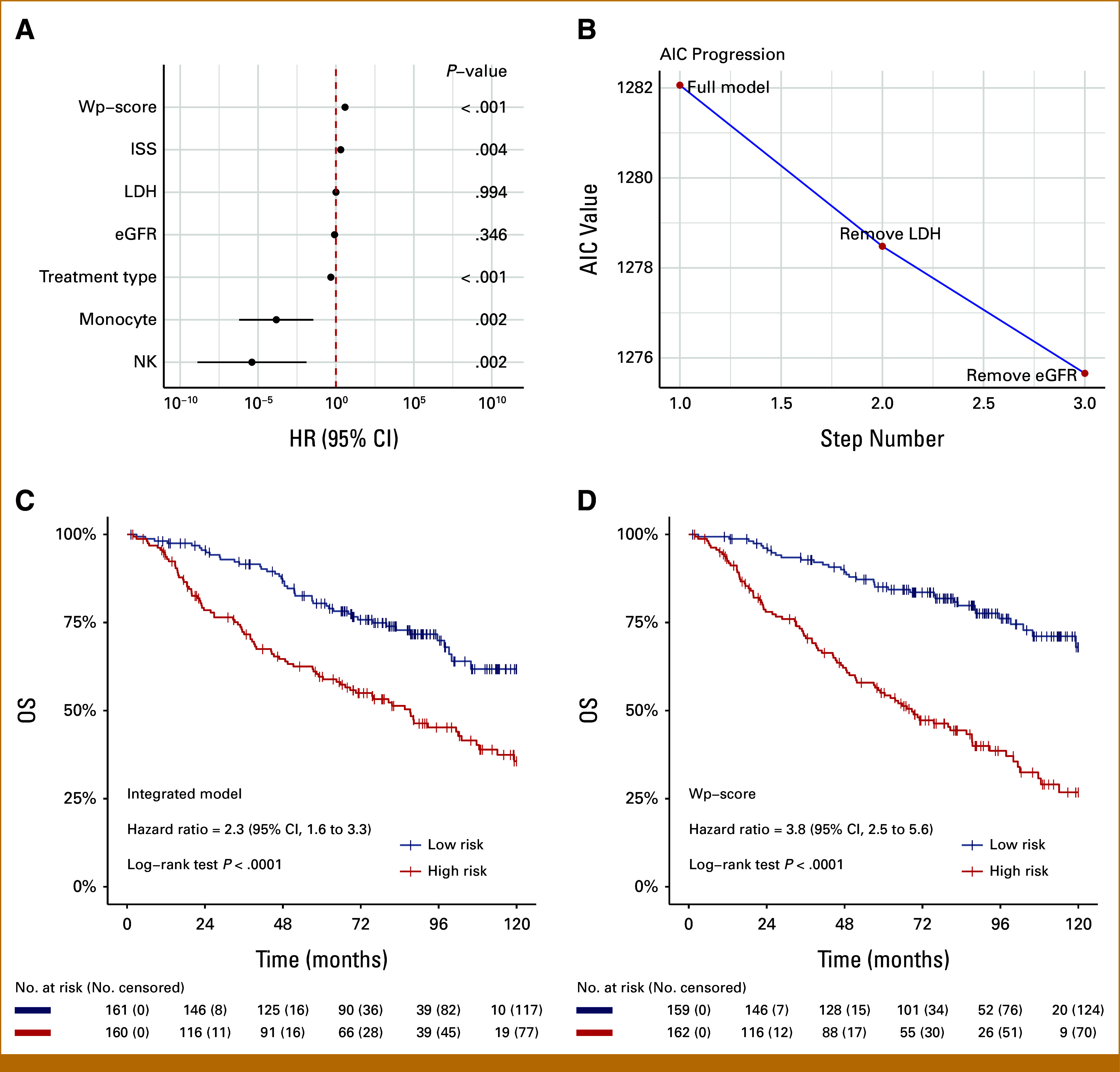
Independent prognostic significance of 5hmC-based wp-score for OS. (A) Cox regression analysis demonstrating that the wp-score remains a significant and independent prognostic variable for OS (*P* < .05); (B) stepwise regression based on the AIC refined the integrated model by retaining key prognostic variables, including the wp-score, ISS, frontline treatment type, and estimated contributions from monocytes and NK cells; (C) performance of the integrated model; (D) the wp-score maintained its strong association with OS. 5hmC, 5-hydroxymethylcytosine; AIC, Akaike information criterion; HR, hazard ratio; ISS, International Staging System; LDH, lactate dehydrogenase; NK T, natural killer T; OS, overall survival; wp-score, weighted prognostic score.

We developed a multivariable Cox regression model (eg, full model), controlling established prognostic factors and estimated cellular contributions from monocytes, NK T cells, and CD8^+^ T cells in the pooled cohort. The wp-score remained a significant, independent predictor of OS (Fig 4A). LASSO Cox regression also retained the wp-score as a key predictor (Data Supplement, Table S4). Stepwise regression on the basis of the AIC identified an integrated model including the wp-score, ISS stage, frontline treatment type, and cellular contribution from monocytes and NK cells (Fig 4B). This model showed that higher risk scores were associated with worse OS (HR, 2.3 [95% CI, 1.6 to 3.3]; Fig 4C). Notably, the wp-score remained significantly associated with OS in the final integrated model (HR, 3.8 [95% CI, 2.5 to 5.6]; Fig 4D), with consistent performance in time-dependent AUC analyses (Data Supplement, Table S5), highlighting its robust and independent prognostic value. In addition, the wp-score showed no significant association with clinical variables (Data Supplement, Table S6) and only modest correlations with immune cell sources (Data Supplement, Table S7).

We found that the wp-score also showed a significant association with PFS (Data Supplement, Fig S5A). A final integrated model was developed using stepwise regression based on AIC, retaining the wp-score, ISS stage, and induction treatment type (Data Supplement, Fig S5B). The resulting risk score showed that higher scores were significantly associated with worse PFS (HR, 2.0 [95% CI, 1.4 to 3.0]; Data Supplement, Fig S5C). Consistent with the OS findings, the wp-score remained significantly associated with PFS (HR, 3.6, 95% CI, 2.4 to 5.6) in the final integrated model (Data Supplement, Fig S5D), further substantiating its prognostic significance independent of other established clinical prognostic factors.

## DISCUSSION

In this prospective study of newly diagnosed patients with MM, we identified an 18-feature 5hmC signature in cfDNA that were associated with OS and PFS. We demonstrated that cfDNA-based 5hmC modifications closely reflected those observed in CD138+ tumor cells, underscoring the biological relevance of these circulating epigenetic markers. Importantly, the wp-score derived from the 18-feature panel at the time of diagnosis was associated with OS and PFS independent of established prognostic factors and predicted survival outcomes over multiple follow-up durations. To our knowledge, this is the first study to delineate genome-wide cfDNA 5hmC profiles in MM and to establish their prognostic value as minimally invasive epigenetic biomarkers.

Our findings provide additional insights into MM pathobiology and risk stratification. The concordance between cfDNA and bone marrow‑derived tumor DNA highlights the potential of cfDNA-based 5hmC profiling as a complementary tool to bone marrow sampling, which is invasive and subject to spatial heterogeneity.^27,28^ Nevertheless, the limited number of paired samples precludes definitive inference regarding tumor specificity. Furthermore, differential analysis across clinical subgroups—defined by disease stages, eGFR, and LDH levels—suggested that cfDNA 5hmC signatures captured disease heterogeneity and reflected underlying biological differences with clinical relevance. Collectively, these data support 5hmC profiling in cfDNA as a promising approach for noninvasive molecular characterization and refined prognostication in MM.

Cellular deconvolution further showed that MM-associated monocytes were the primary source of cfDNA in MM. Monocyte-derived myeloid suppressor cells and tumor-associated macrophages are known to promote plasma cell proliferation within the bone marrow.^45,46^ Elevated peripheral monocyte counts at diagnosis and during follow-up have been associated with inferior OS in MM,^47^ consistent with our observations. Moreover, we found differential contributions of NK cells and CD8^+^ T cells to cfDNA at diagnosis between patients at risk and those who later died. Increased NK-derived cfDNA in patients with favorable outcomes may reflect a more active innate immune response and improved tumor surveillance,^48^ whereas greater CD8^+^ T-cell contribution in diseased patients may indicate a reactive but ineffective adaptive immune activation against aggressive disease,^49^ suggesting that cellular origins of cfDNA encapsulated the immune landscape. Furthermore, pathway enrichment analysis revealed important involvement of several biologically relevant signaling cascades, notably calcium signaling, which regulates proliferation and apoptosis and is frequently dysregulated in MM.^50,51^ However, because circulating cfDNA originates from multiple cellular compartments, pathway enrichment findings should be interpreted cautiously, as signals may reflect both tumor-intrinsic biology and host immune or stromal contributions.

In this study, our 5hmC-based wp-score demonstrated consistent prognostic accuracy for OS and PFS. The model components included in the final panel involve multiple biological processes, including immune regulation, transcriptional control, and cellular metabolism. For example, *SPEN* functions as a transcriptional corepressor with roles in epigenetic regulation and immune-related signaling pathways,^52^ *GTF2I* plays an important role in lymphocyte biology,^53^ and *PPARD* is involved in metabolic regulation and tumor progression in cancer.^54^ Higher wp-scores consistently identified patients with inferior outcomes, independent of demographic and clinical prognostic factors, reflecting the model's potential clinical utility. However, because the wp-score was developed with OS as the primary end point, its predictive performance for PFS may be comparatively weaker. An integrated model combining the wp-score with established prognostic factors and cfDNA cellular contributions further improved prognostic performance. Notably, the wp-score at diagnosis retained prognostic significance independent of clinical prognostic factors, supporting its potential value for risk stratification.

This study has several strengths, including the rigorous confirmation of MM diagnosis using the IMWG criteria; validation of clinical outcomes; a prospective study design; and the application of 5hmC-Seal, a cutting-edge technology for profiling 5hmC modifications in clinical samples. There are also limitations. First, our study was restricted to baseline 5hmC profiles at the time of diagnosis. While we demonstrated the prognostic significance and predictive accuracy of wp-scores for OS and PFS, we were unable to assess the temporal dynamics of 5hmC modifications throughout the course of treatment or after treatment. Independent external validation and temporally distinct patient population will be important to establish clinical utility of this noninvasive approach within contemporary MM risk stratification amid evolving therapeutic landscapes. Second, age-related clonal hematopoiesis may influence circulating epigenetic signals and therefore represents a potential source of variability in cfDNA. In addition, the prognostic 5hmC signature identified likely represents a composite circulating epigenomic signal reflecting both tumor-derived and host-biology‑related processes. Unfortunately, measures such as ctDNA fraction or marrow plasma cell percentage were not available, precluding formal evaluation of whether the wp-score provides prognostic information beyond tumor burden. Third, the analysis was performed using the hg19 reference genome. Future analyses aligned to the hg38 reference genome may further improve genomic annotation.

In conclusion, this study suggests that cfDNA-derived 5hmC profiling captures features of the tumor-associated epigenetic landscape and provides prognostic information in newly diagnosed MM. We identified an 18-feature wp-score associated with OS and PFS independent of established clinical prognostic factors. These findings support further investigation and independent validation of noninvasive epigenomic biomarkers within contemporary MM risk stratification frameworks as therapeutic approaches continue to evolve.

## Data Availability

A data sharing statement provided by the authors is available with this article at DOI https://doi.org/10.1200/PO-25-01121. The summarized gene-level 5hmC profiles are available at the NCBI GEO under accession number GSE186351.
